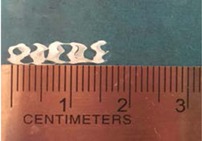# Fabrication and Optimal Design of Biodegradable Polymeric Stents for Aneurysms Treatments

**DOI:** 10.3390/jfb8010008

**Published:** 2017-02-28

**Authors:** Xue Han, Xia Wu, Michael Kelly, Xiongbiao Chen

**Affiliations:** 1 Department of Mechanical Engineering, University of Saskatchewan, Saskatoon, SK S7N 5A9, Canada; xbc719@mail.usask.com; 2 Division of Biomedical Engineering, University of Saskatchewan, Saskatoon, SK S7N 5A9, Canada; wuxiayunxin@126.com; 3 Department of Neurosurgery, University of Saskatchewan, Saskatoon, SK S7N 5A9, Canada; m.kelly@usask.ca

**Keywords:** biodegradable polymeric stents, fabrication, radial stiffness, simulation, optimization

## Abstract

An aneurysm is a balloon-like bulge in the wall of blood vessels, occurring in major arteries of the heart and brain. Biodegradable polymeric stent-assisted coiling is expected to be the ideal treatment of wide-neck complex aneurysms. This paper presents the development of methods to fabricate and optimally design biodegradable polymeric stents for aneurysms treatment. Firstly, a dispensing-based rapid prototyping (DBRP) system was developed to fabricate coil and zigzag structures of biodegradable polymeric stents. Then, compression testing was carried out to characterize the radial deformation of the stents fabricated with the coil or zigzag structure. The results illustrated the stent with a zigzag structure has a stronger radial stiffness than the one with a coil structure. On this basis, the stent with a zigzag structure was chosen for the development of a finite element model for simulating the real compression tests. The result showed the finite element model of biodegradable polymeric stents is acceptable within a range of radial deformation around 20%. Furthermore, the optimization of the zigzag structure was performed with ANSYS DesignXplorer, and the results indicated that the total deformation could be decreased by 35.7% by optimizing the structure parameters, which would represent a significant advance of the radial stiffness of biodegradable polymeric stents.

## 1. Introduction

An aneurysm is a balloon-like bulge in the wall of blood vessels, occurring in major arteries of the heart (aortic aneurysm) and brain (cerebral aneurysm). In America each year, aortic aneurysms cause over 13,000 deaths [1], while cerebral aneurysms contribute to approximately 30,000 people suffering a rupture [2]. When an intracranial aneurysm ruptures, it may bleed into the subarachnoid space and result in a subarachnoid hemorrhage, with a mortality rate of 25%–50% [2]. Endovascular treatment (EVT) is a pharmacological and/or mechanical intervention for treating arterial disease. EVT by selective coiling is considered one of the main treatments for intracranial aneurysms, which is limited with respect to the treatment of wide-necked and/or large aneurysms, and recanalization would occur after the EVT treatment.

To circumvent these limitations, a number of new devices have been developed over the last several decades [3]. Among them, self-expandable stents made from metals and/or biodegradable polymeric materials are shown promising by providing scaffolding support in the blood vessel. Metallic stents have been considered the gold standard in terms of treatment of complex intracranial aneurysms. Recently, biodegradable polymeric stents (BDS) have been emerging and demonstrating advantages over permanent metallic stents [4]. First, the option of BDS may alleviate patient concerns over permanent implants, especially with respect to the possibility of late stent thrombosis and in-stent stenosis [5]. Second, BDS are able to help control drug release and facilitate the reduction of in-stent neointimal formation, thus minimizing the circumstance of restenosis [6]. With the degradation of the stents, only is a healed arterial vessel left behind. [7]. There are a number of biodegradable polymeric stents reported in the literature [8,9,10] with varying degrees of successes, but leaving several issues and/or limitations to be addressed. Most biodegradable polymeric stents are made with a zigzag structure, but there is no any optimal design method utilized. This often causes weaker mechanical properties compared to native arterial vessels and result in early recoil post implantation [8]. In addition, the conventional methods of fabricating stents are typically to braid monofilaments of biodegradable polymer into a tubular structure [9,10], thus being time-consuming, expensive, and lacking precise control over the stent microstructure. Therefore, the conventional methods of fabricating stents result in poor repeatability and limit the design improvements that can be made. As an emerging fabrication technique, the dispensing-based rapid prototyping (DBRP) technique allows for more accurate control over the scaffold microstructure, thus facilitating the fabrication of stents as designed [11,12].

This study is aimed at developing methods for fabrication and optimal design of biodegradable polymeric stents for treating aneurysms. Firstly, a method was developed to fabricate biodegradable polymeric stents by using the DBRP technique. Then, compression testing was carried out to characterize the radial deformation of the stents fabricated. Furthermore, a simulation of compression test was pursued based on finite element model; and a study on optimizing the structure of biodegradable polymeric stents though the ANSYS Design Xplorer was conducted.

## 2. Materials and Methods

### 2.1. Material Preparation

Polycaprolactone (PCL) powder (M.W. 50,000) was purchased from Polysciences, Inc. (Warrington, FL, USA). A solvent of chloroform was used, and the concentration of polymer was prepared varying from 50% PCL to 70% PCL under a chemical hood. Specifically, the polymer was weighted at 5 g, 6 g, and 7 g, respectively, for 50%, 60%, and 70% concentration solutions and then placed into a glass jar, which was then supplemented with 10 mL of chloroform and stirred until a homogeneous solution was obtained. All solutions were prepared at room temperature.

### 2.2. Fabrication Method

**Figure 1:**
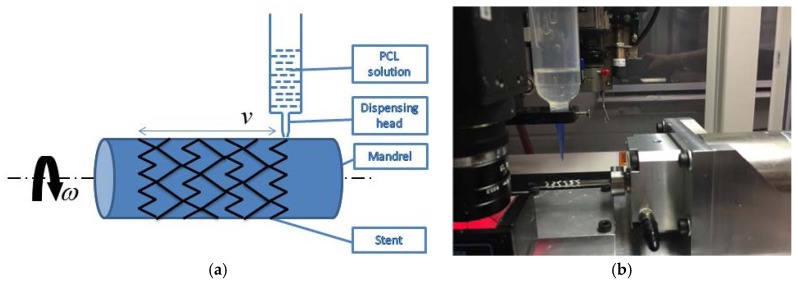
Picture of fabrication setting. (**a**) Schematic of fabrication of biodegradable stents. (**b**) Fabrication system.

In this study, a fluid dispensing system (C0720M, Asymtek, Carlsbad, NM, USA) was used for dispensing biomaterials, as shown in Figure 1. A motorized cylindrical substrate was utilized, where a replaceable mandrel was connected to the shaft of an electric motor, as such the mandrel can be rotated in a controlled manner; and the biomaterial solution was dispensed onto the surface of the mandrel as it rotates, forming stents with a 3D cylindrical structure. On this dispensing system, the biodegradable polymeric stents can be manufactured with a strand diameter down to 0.1 mm. During the fabrication process, the flow rate of solution dispensed and the moving speed of the dispensing head needed to be appropriately determined and used. Notably, the flow rate was affected by the concentration of solutions, needle size, and air pressure applied to the syringe [11,12]. Additionally, the angular velocity of the mandrel and the moving speed of the dispensing head needed to be coordinated, depending on the design of stents.

**Figure 2:**
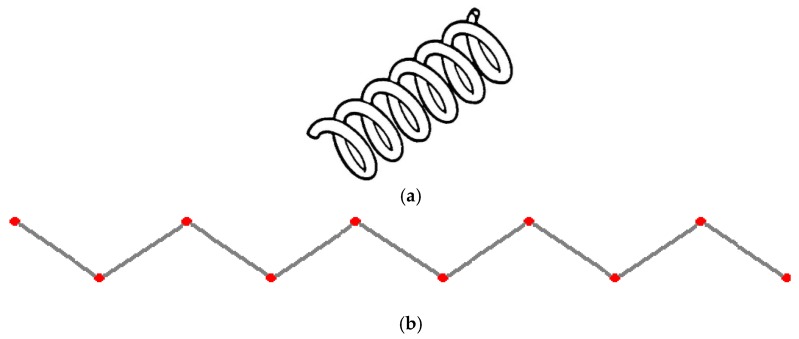
Structures of biodegradable polymeric stents. (**a**) Coil structure. (**b**) Zigzag structure.

Biodegradable polymeric stents with the coil and zigzag structures (Figure 2) were fabricated, with the fabrication parameters including air pressure, dispensing head moving speed, needle size, mandrel rotating speed, and dispensing head height detailed later on.

### 2.3. Characterization of Radial Stiffness

The radial stiffness, a measurement of the radial rigidity of the biodegradable polymeric stents, is of importance for the reduction of target vessel restenosis, as the radial stiffness can present the extent to resist radial deformation of biodegradable polymeric stents in response to an applied force [13]. The compression test is an efficient method to investigate the radial stiffness of biodegradable polymeric stents by examining the deformation behaviors of biodegradable polymeric stents under loads. This test was intended to provide a compression comparison of radial deformation among biodegradable polymeric stents including coil and zigzag structure fabricated. Specifically, the biodegradable polymeric stents were compressed, and the displacement at various loads were recorded.

**Figure 3:**
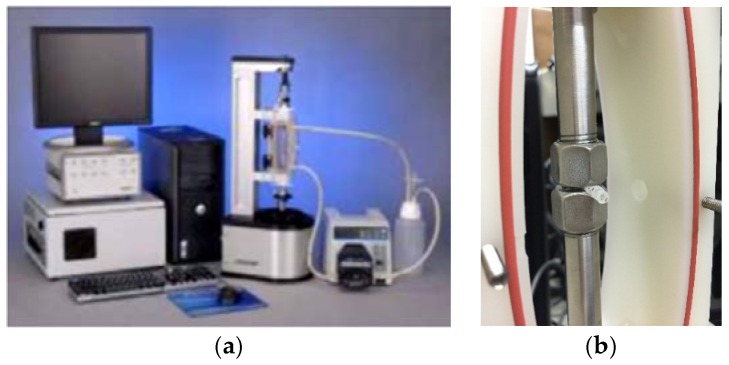
Compression tests for biodegradable polymeric stents. (**a**) Bose ElectroForce Biodynamic testing system. (**b**) Loading plates for specimen.

An advanced design of the Bose Dynamic testing platform to evaluate the deformation of biodegradable polymeric stents was employed, which can provide an accurate characterization of biomaterials and biological samples within a sterile cell culture media environment. During the test, the biodegradable polymeric stents with a zigzag structure and coil structure were compressed, and displacements versus the applied loads were recorded. In this experiment, the compression rate was set at 0.1 mm/s (Figure 3).

The diameters of both coil and zigzag structures of biodegradable polymeric stents are the same (i.e., 3.4 mm). The forces required to compress the stents radially by 10% (0.34 mm), 20% (0.68 mm), 30% (1.02 mm), and 40% (1.36 mm) were measured. Nine groups of biodegradable polymeric stents need to be tested, and there are four stent samples in each group (Table 1). The letters from A to G are used to denote the zigzag biodegradable polymeric stents, which were fabricated by different fabrication parameters and varying concentration solutions. The concentration of the solution of biomaterials is the same (70%) from A to E, while the fabrication parameter (the speed of dispensing head) is different (0.85 inch/s, 0.7 inch/s, 0.9 inch/s, 1.1 inch/s, and 1.3 inch/s, respectively). F and G denote the 60% and 50% zigzag biodegradable polymeric stents when the speed of dispensing head is at 0.7 inch/s. Coil A and Coil B stand for coil structure of biodegradable polymeric stents at the concentration of 50% and 70%, respectively.

Under the circumstance of the optimal parameters not known, the groups (B, F, and G) were fabricated first to test the proper concentration of material. Based on the result, 70% concentration of PCL and 0.41 mm diameter of dispensing needle are best for fabrication parameters. Another experiment (Groups of A, C, D and E) was then designed with a different dispensing speed to obtain a stronger structure of zigzag stent.

### 2.4. Compression Test Simulation

The simulation of compression test of biodegradable polymeric stents was performed by a static structural analysis, which determined the displacements, stresses, strains, and forces in the stent structure caused by loads, under the assumption that the inertia and damping effects are ignored [14]. Additionally, the loads and the structure’s response are assumed to vary slowly with respect to time. The results of the simulation were given in terms of the load-displacement curve for the biodegradable polymeric stents. The linear elastic material behavior is assumed, and small deflection theory is used. [K] is the stiffness matrix, and {F} is the load statically applied. Specifically, the displacements {x} was solved from the following equation [15]:

[K]{x} = {F}.(1)

For the biodegradable polymeric stents fabricated by the dispensing-based rapid prototyping machine, the thickness of the strut is 0.41 mm and the diameter of the stent is 3.4 mm. The geometrical information was specified in the Pro/Engineer software and then used for simulation in the ANSYS Workbench.

**Figure 4:**
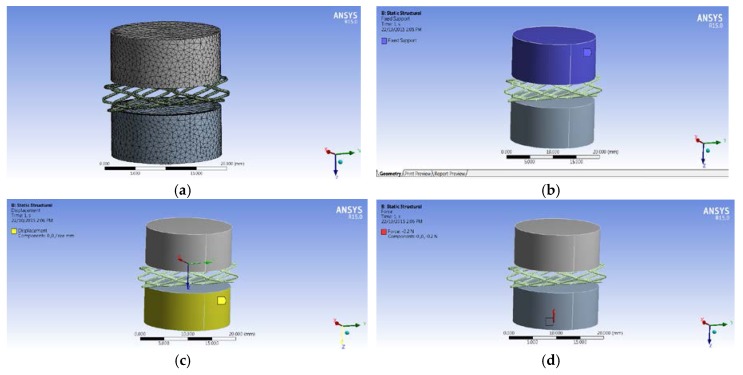
Simulation of compression test. (**a**) Mesh. (**b**) Fixed support on the upper plate. (**c**) Fixed support on the upper plate. (**d**) Force applied on the lower plate.

The Engineering Data Manager in ANSYS Workbench provides a powerful tool for defining, organizing, and storing material properties. A great variety of material models are available in the Toolbox menu, and material data can be stored in libraries that can be accessed in any project. The biodegradable polymeric stent is made of PCL while the compression plates are made of structural steel. The material properties [16] defined are density, Young’s Modulus, and Poisson’s Ratio. The Mesh Tool in ANSYS Workbench provides an accessible path to many of the most common mesh controls. The Tetrahedral meshing method was used for meshing biodegradable polymeric stents, and multiple triangular surface meshing algorithms were applied to ensure high quality (Figure 4a).

Based on the equipment used in the compression test, the upper plate was fixed, and the plate below could be moved with the force applied. Therefore, the fixed support was used on the top plate (Figure 4b), and the displacement of the *X* and *Y* directions on the lower plate was zero, while the displacement of the *Z* direction was free (Figure 4c). Additionally, the initial force is −0.2 N with the opposite *Z* direction (Figure 4d).

### 2.5. Optimal Design of Stents

ANSYS DesignXplorer is an optimization tool, which works under the ANSYS Workbench environment [17]. ANSYS DesignXplorer interacts with all ANSYS Workbench platform components and leads CAD packages such as Pro/ENGINEER directly so that design changes to the design database can be made quickly [17]. Therefore, the simulation model of the biodegradable polymeric stents built can be used for parametric optimization. The instantaneous feedback on design modifications provided by ANSYS DesignXplorer dramatically decreases the number of design iterations. The goal here is to determine the parameters of the biodegradable polymeric stent that offer the minimum radial deformations under a given force. With the design of experiments, several combinations of the parameters are created as “design points”.

**Figure 5:**
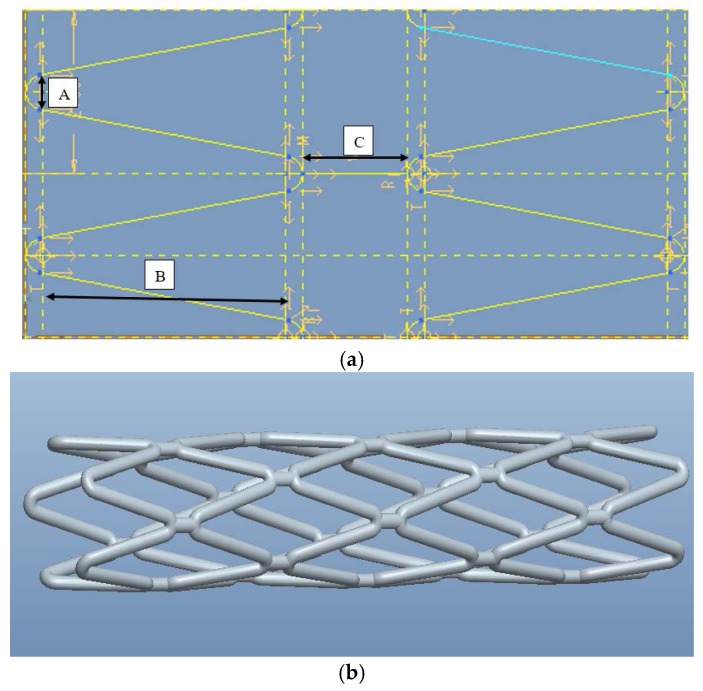
Model of biodegradable polymeric stents built in Pro/Engineer. (**a**) Parameters for each cell of zigzag biodegradable stents. (**b**) 3D model of zigzag biodegradable polymeric stent.

There are three parameters to represent a zigzag cell in the axial direction (Figure 5a). The one denoted by A in Figure 5a is the diameter of curvature at the apex that is tangential to the line on each side in the circumferential direction. As the diameter of curvature changes, the angle between the two lines will be altered. The one denoted by B is the vertical length of the line tangential with the semi-circle. The one denoted by C is the distance between the two apexes of the semi-circles. Different designs of stents (Figure 5b) can be obtained by changing the parameter values. Initially, A was set as 0.25 mm; B as 2.8 mm; C as 0.8 mm.

For optimization, the input parameters are geometric parameters (A, B and C) and the output parameter is the deformation of the biodegradable polymeric stent. Each combination of input parameters used gives a design point and the simulation is to examine the corresponding output response for a given design point.

## 3. Results and Discussion

### 3.1. Biodegradable Polymeric Stents Fabricated by DBRP Technique

#### 3.1.1. Coil Structure with Different Concentration of PCL Solution

For the stent fabrication, the working parameters can be broadly divided into six categories, which are the concentration of PCL solution, air pressure, dispensing height, dispensing speed, mandrel rotating speed, and diameter of the needle of dispensing head (Table 2). In this experiment, the parameter including air pressure (30 psi), dispensing height (1.74 inch), dispensing speed (0.7 inch/s), and mandrel rotating speed (402 rpm) were kept the same.

The concentration of PCL solution plays a pivotal role in the coil formation during the fabrication process. Three levels from low to high were utilized in this study. As the low concentration leads to the low viscosity, the smaller diameter of the needle for the dispensing head had to be used so that the struts could solidify on the mandrel and not to flow out of the mandrel. A 70% PCL solution worked well with the 0.41 mm diameter of the needle for the dispensing head, while the 60% PCL solution could not form a round strut through a 0.41 mm diameter of the needle for the dispensing head because of its low viscosity. Air pressure and dispensing height also have a significant effect on the flow rate of the PCL solution within the syringe. If the flow rate is too high or too low, the bead strut from the syringe will come out rather than a smooth one. In this study, the air pressure and dispensing height were adjusted at 30 psi and 1.74 inches respectively for a stable strut. For the coil structure, the mandrel rotating speed had to be relatively high (402 rpm) and the speed of dispensing head was moderate (0.7 inches/s) to keep an average pitch of the coil structure stents.

#### 3.1.2. Zigzag Structure with Different Concentration of PCL Solution

Three concentrations of PCL solutions were used for fabricating zigzag stents (Table 3). In this experiment, the parameters including air pressure (30 psi), dispensing height (1.74 inch), dispensing speed (0.7 inch/s), and mandrel rotating speed (232 rpm) were kept the same.

It was found that 70% of PCL solution worked well with the 0.41 mm diameter of the needle, while the 50% PCL solution for 0.25 mm diameter of the needle. The air pressure and dispensing height were the same as the values for fabricating the coil structure. The mandrel rotating speed (232 rpm) in fabricating zigzag structure was set slower than the one for making coil structure because more time was needed for the dispensing head to move forward and back to form a zigzag pattern. The numbers of the zigzag in one row determine the mandrel rotating speed and the speed of dispensing head, which means that the low mandrel rotating speed allows more forward and back movements

#### 3.1.3. Zigzag Structure Made of 70% PCL Solution

In order to obtain a zigzag structure with better mechanical property stents, another experiment was carried out to investigate the parameters of fabricating the 70% PCL solution.

The speed of dispensing head is of importance for forming a zigzag structure of biodegradable polymeric stents, which was set from 0.7 to 1.3 inch/s in this experiment, while other parameters were kept the same value (Table 4). By visual observation, it was found that, if the speed of dispensing head was at 0.85 inches/s, the zigzag structure of biodegradable polymeric stent fabricated looked better than others. The property of radial deformation of all of the biodegradable polymeric stents was examined by a compression test. The parameters were set as follows: the concentration of PCL solution is 70%, air pressure 30 psi, dispensing height 1.74 inch, diameter of dispensing head 0.41 mm, and mandrel rotating speed 402 rpm.

### 3.2. Characterization of Biodegradable Polymeric Stents for Coil and Zigzag Structures

**Figure 6:**
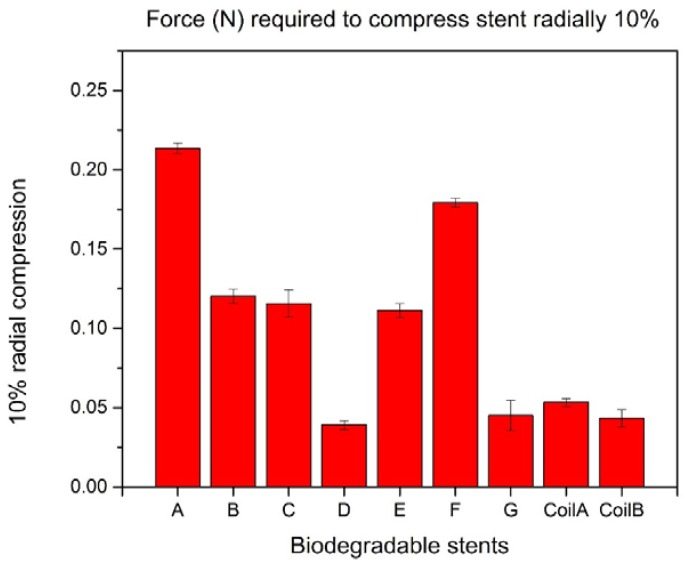
10% radial compression of biodegradable polymeric stents.

All the biodegradable polymeric stents were compressed in a similar elliptical shape configuration. For the forces required to compress the biodegradable polymeric stents radially by 10%, the largest force was 0.214 N for Group A, and the second one was 0.179 N for Group F. The result means that the zigzag structure in Group A can bear more compression forces than other groups and indicates that the zigzag structure in Group A is the strongest mechanically (Figure 6).

**Figure 7:**
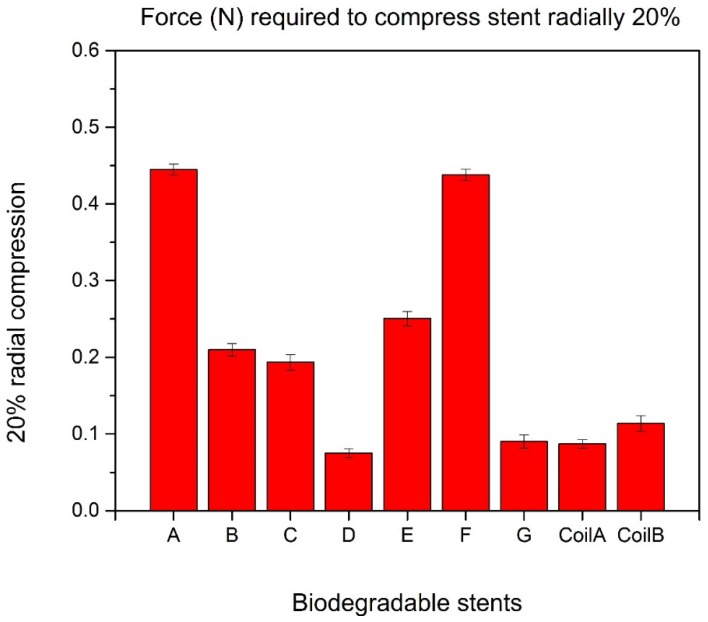
20% radial compression of biodegradable polymeric stents.

There is no significant difference between the largest force and the second one when compressing the biodegradable polymeric stents radially 20%, which are 0.445 N in Group A and 0.438 N in Group F, respectively (Figure 7).

**Figure 8:**
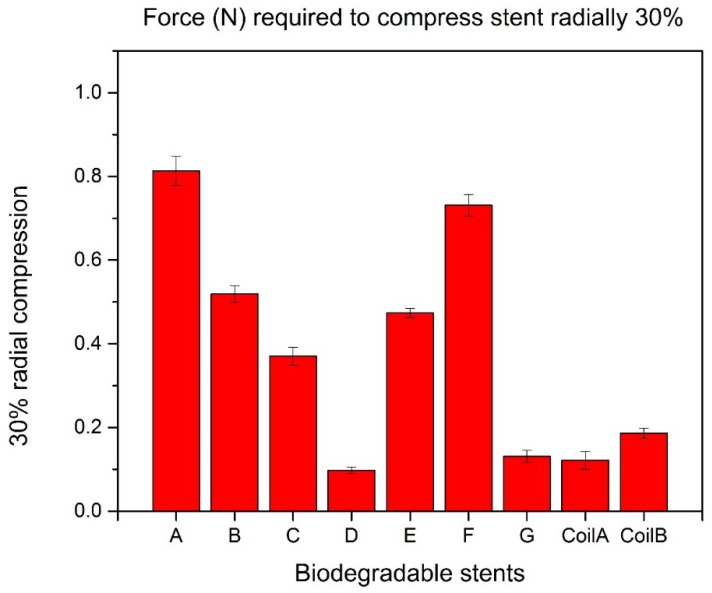
30% radial compression of biodegradable polymeric stents.

The largest force can compress biodegradable polymeric stents radially 30% is 0.813 N in Group A while the second one is 0.713 N in Group F (Figure 8).

**Figure 9:**
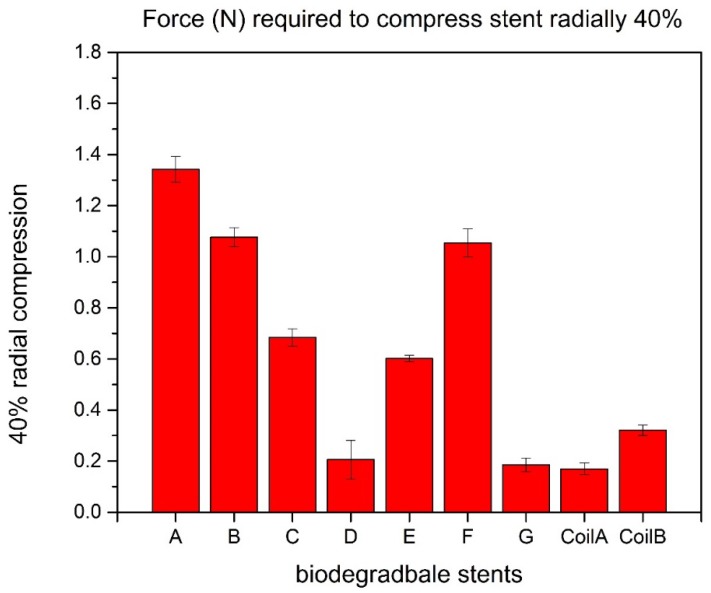
40% radial compression of biodegradable polymeric stents.

To compress biodegradable polymeric stents radially 40%, the force in Group A is 1.342 N, with a 27% larger than the second one (Figure 9).

Based on the experiment results, the zigzag biodegradable polymeric stents in Group A and Group F can bear more forces than other stents in other groups. Both of the biodegradable polymeric stents in Group A and Group F have zigzag structure. The viscosity of 60% concentration PCL solution in Group F is lower than the viscosity of 70% concentration PCL solution used in Group A, due to the same diameter (0.41 mm) of dispensing heads used in those two groups for dispensing the struts, which leads to all the struts at the apex of zigzag structure cross-linked in Group F; thus, an increase of radial stiffness obtained in Group F. The parameters for fabricating biodegradable polymeric stents in Group A can make non-cross-linked struts with a high radial stiffness. Additionally, the biodegradable polymeric stents with a zigzag structure are much stronger than biodegradable polymeric stents with a coil structure. The main reason is that a polymer fiber coil stent cannot keep its original shape well with lower mechanical strength than zigzag stents [18]. Dr. Satio examined the mechanical strength of biodegradable polymer-knitted stent made of poly-_L_-lactic acid and silicone in vitro. For diameters between 4 and 6 mm, the deformation rate of the silicone stent was lower than that of the PLLA stent. However, the mechanical strength of PLLA stents increased as a function of their diameter. When the radial compression was 10%, 20%, 30%, and 40%, the applied force was approximately 0.11 N, 0.25 N, 0.42 N, and 0.61 N respectively. In comparison to the PLLA stent by Dr. Satio, the PCL stents made in our lab are much stronger. The applied force was approximately 0.21 N, 0.44 N, 0.82 N, and 1.35 N respectively for 10%, 20%, 30%, and 40% radial compression. [19].

### 3.3. Comparison between Real Compression Test and Simulation

**Figure 10:**
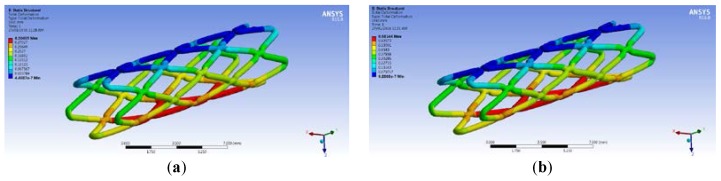
Deformation of compressing biodegradable polymeric stents radially 10% (**a**) and 20% (**b**).

A model for simulation was built based on the zigzag structure of biodegradable polymeric stents in Group A. The forces required to compress biodegradable polymeric stents in Group A radially 10% (0.342 mm) is 0.199 N. When the force is 0.199 N applied in the simulation, the total deformation of the biodegradable polymeric stent is 0.304 mm (Figure 10a), the error is 11.11%. The force compressing biodegradable polymeric stents radially 20% is then applied on the biodegradable polymeric stent in the simulation, and the total deformation of the biodegradable polymeric stent are 0.681 mm, and the error is 0.29% compared with the real compression test (0.679 mm in Figure 10b).

**Figure 11:**
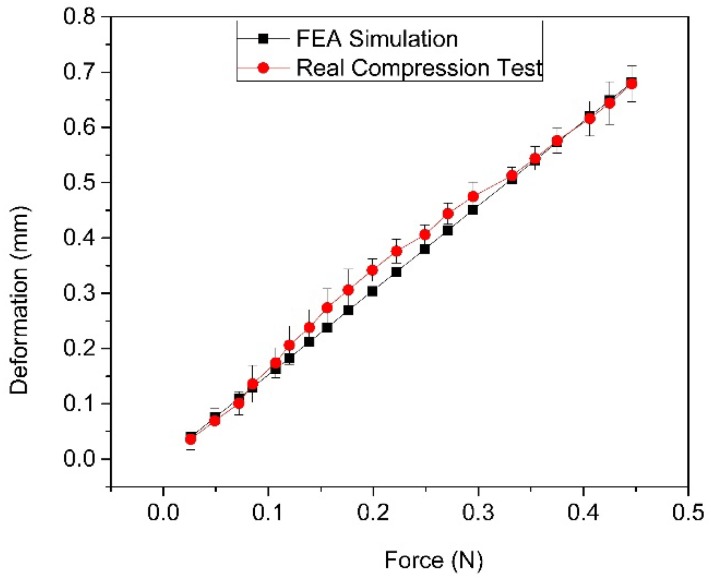
Total deformation and force relationship comparison between FEA and real compression test.

The deformation comparison of biodegradable polymeric stents between simulation and the actual compression test is presented in Figure 11. The relationship between force and deformation is linear in the simulation of ANSYS Workbench, while in the actual compression test the relationship between force and deformation is slightly non-linear. The error based on the comparison results (where the maximum error is 13.14%) is insignificant, indicating that the finite element model is acceptable within the range of around 20% radial deformation.

The deformation comparison of biodegradable polymeric stents between the simulation and actual compression test shows that the finite element model for simulating the compression test can be accepted within the range of around 20% radial deformation. The simulation model of biodegradable polymeric stent was further used for optimization of stents as follows.

### 3.4. Optimization of Biodegradable Polymeric Stents

**Figure 12:**
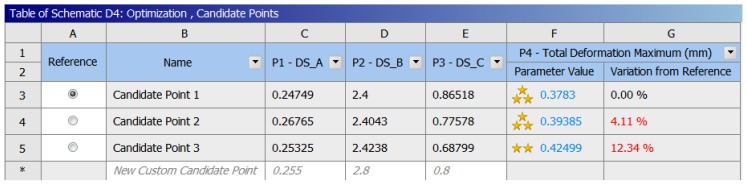
Optimization results of biodegradable polymeric stents.

The goal here is to determine the parameters of the biodegradable polymeric stent that offer the minimum radial deformations under the given force. With the design of experiments, several combinations of the input parameters are created as “design points.” By running the analysis for each design point and recording the output, a mathematical formula was fitted to the data. Finally, the input parameters are determined from this formula with a significant effort on the outputs. The optimization results are shown with ratings (the more stars, the better). Candidate Point 1 is in this case the best choice, among the 10,000 sampled points, and the total deformation of biodegradable polymeric stents will be 0.3783 mm (Figure 12). Compared with the initial total deformation (0.5887 mm) when applying the force (−0.4 N) to compress the biodegradable polymeric stent, it decreases by 35.7%, which makes a significant contribution to increasing the mechanical property. The parameters obtained from the optimization are 0.247 mm for Parameter A, 2.4 mm for Parameter B, and 0.865 mm for Parameter C.

## 4. Conclusions

This paper presents the process of fabricating biodegradable polymeric stents, and the results show that the dispensing-based rapid prototyping method is a promising technique for fabricating biodegradable polymeric stents. This method is controllable and able to fabricate more various structures of biodegradable polymeric stents, including the coil and zigzag structures. The compression test results indicate that the radial stiffness of zigzag biodegradable polymeric stents is better than the coil stents. Moreover, the results of optimization of biodegradable polymeric stents present that the total deformation can be decreased by 35.7% (from 0.589 mm to 0.378 mm) by modifying the parameters of the geometry, which makes a significant contribution to increasing the radial stiffness of biodegradable polymeric stents.

## Figures and Tables

**Table 1:** Nine groups of biodegradable polymeric stents for compression test.

Label	Concentration	Speed of Dispensing Head
A	70%	0.85
B	70%	0.7
C	70%	0.9
D	70%	1.1
E	70%	1.3
F	60%	0.7
G	50%	0.7
Coil A	50%	0.7
Coil B	70%	0.7

**Table 2:** Fabrication parameters for coil structure of biodegradable polymeric stents.

Label	Concentration of PCL Solution	Diameter of the Needle (mm)	Samples
Coil 50	50% PCL	0.25	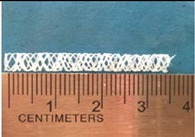
Coil 60	60% PCL	0.41	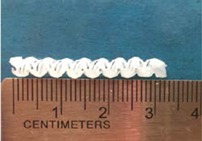
Coil 70	70% PCL	0.41	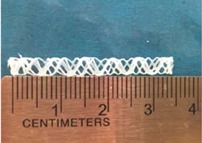

**Table 3:** Fabrication parameters for zigzag biodegradable polymeric stents.

Label	Concentration of PCL solution	Diameter of the Needle (mm)	Samples
Zigzag 50	50% PCL	0.25	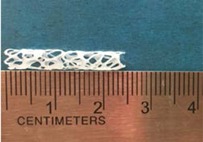
Zigzag 60	60% PCL	0.41	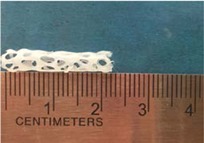
Zigzag 70	70% PCL	0.41	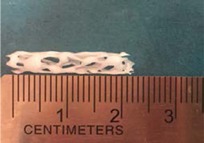

**Table 4:** Fabrication parameters for 70% PCL of zigzag biodegradable polymeric stents.

Label	Dispensing Speed (Inch/s)	Samples
Zigzag 70-0.7	0.7	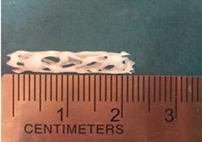
Zigzag 70-0.85	0.85	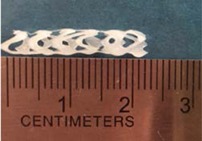
Zigzag 70-0.9	0.9	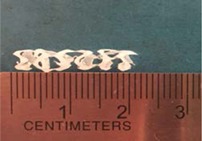
Zigzag 70-1.1	1.1	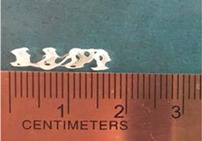
Zigzag 70-1.3	1.3